# Exploring the Interaction of G-quadruplex Binders with a (3 + 1) Hybrid G-quadruplex Forming Sequence within the PARP1 Gene Promoter Region

**DOI:** 10.3390/molecules27154792

**Published:** 2022-07-26

**Authors:** Stefania Mazzini, Salvatore Princiotto, Roberto Artali, Loana Musso, Anna Aviñó, Ramon Eritja, Raimundo Gargallo, Sabrina Dallavalle

**Affiliations:** 1 Department of Food, Environmental and Nutritional Sciences (DEFENS), University of Milan, 20133 Milan, Italy; salvatore.princiotto@unimi.it (S.P.); loana.musso@unimi.it (L.M.); sabrina.dallavalle@unimi.it (S.D.); 2 Center for Research in Biosciences & Health Technologies (CBIOS), Universidade Lusófona de Humanidades e Tecnologias, Campo Grande 376, 1749-024 Lisbon, Portugal; 3 Scientia Advice di Roberto Artali, 20832 Desio, Italy; roberto.artali@scientia-advice.com; 4 Institute for Advanced Chemistry of Catalonia (IQAC), CSIC, Networking Center on Bioengineering, Biomaterials and Nanomedicine (CIBER-BBN), ISCIII, 08034 Barcelona, Spain; aaagma@cid.csic.es (A.A.); recgma@cid.csic.es (R.E.); 5 Department of Chemical Engineering and Analytical Chemistry, University of Barcelona, 08028 Barcelona, Spain; raimon_gargallo@ub.edu; 6 National Institute of Fundamental Studies, Kandy 20000, Sri Lanka

**Keywords:** G-quadruplex, PARP1 promoter, stabilizing ligand, NMR, circular dichroism, fluorescence, molecular modeling

## Abstract

The enzyme PARP1 is an attractive target for cancer therapy, as it is involved in DNA repair processes. Several PARP1 inhibitors have been approved for clinical treatments. However, the rapid outbreak of resistance is seriously threatening the efficacy of these compounds, and alternative strategies are required to selectively regulate PARP1 activity. A noncanonical G-quadruplex-forming sequence within the PARP1 promoter was recently identified. In this study, we explore the interaction of known G-quadruplex binders with the G-quadruplex structure found in the PARP gene promoter region. The results obtained by NMR, CD, and fluorescence titration, also confirmed by molecular modeling studies, demonstrate a variety of different binding modes with small stabilization of the G-quadruplex sequence located at the PARP1 promoter. Surprisingly, only pyridostatin produces a strong stabilization of the G-quadruplex-forming sequence. This evidence makes the identification of a proper (3+1) stabilizing ligand a challenging goal for further investigation.

## 1. Introduction

Poly (ADP-ribose) polymerase-1 (PARP1) is a nuclear enzyme involved in DNA repair processes [1,2]. The enzyme repairs breaks in single-strand DNA through a base excision repair pathway. Additionally, PARP1 is implicated in other cellular processes, such as transcriptional regulation, chromatin remodeling, cell signaling and cell death [3,4].

PARP1 inhibition causes the so-called “synthetic lethality” in tumor cells with defective homologous recombination pathways and sensitizes the tumor cells to DNA-damaging chemotherapies, including multiple chemotherapy or radiotherapy approaches, which remain the backbone of treatment for most cancer patients [5]. Consequently, PARP1 has emerged as an attractive target for cancer therapy. Several PARP1 inhibitors have been successfully developed, including Olaparib, Veliparib, Rucaparib, Talazoparib and Niraparib [6,7].

However, the emergence of resistance to the PARP1 inhibitors, mediated by multiple molecular mechanisms, has generated the need for alternative approaches to interfere with PARP1 activity.

In this context, an unexplored and promising approach could be related to a transcriptional repression of the enzyme. Recently, Chambers et al. investigated the promoter region of the PARP1 gene and identified noncanonical G-quadruplex-forming sequences by genome-mapping experiments [8].

G-quadruplexes are four-stranded structures formed by G-rich nucleic acids comprising a stack of multiple guanine(G)-tetrads [9]. A wide variety of G-quadruplexes were elucidated. These structures were extensively associated with cancer, playing an important role in telomere maintenance and in controlling the expression of several oncogenes and tumor suppressors [10,11]. For these reasons, the ligand-mediated structure modulation of G4-DNA in gene promoter regions may represent an approach to control deleterious gene expression. Both the DNA and RNA G-quadruplex structures are being extensively investigated as potential therapeutic targets for small-molecule G-quadruplexes binders [12].

**Figure 1:**
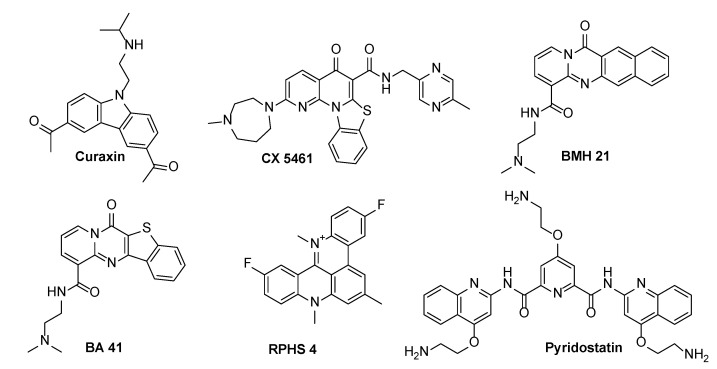
Structures of selected G-quadruplex binders.

Notably, the topology and loop conformation of the G4-DNA structures are known to be highly polymorphic, with the folding conditions and the nature of the loop sequences determining the overall topology. Small molecules end-stacking with G4-DNA include planar aromatic surfaces that mimic the large planar surface of the G-quadruplex. As end-stacking only requires a G-tetrad (i.e., a planar aromatic surface) to stack with, the binding of these molecules may not require a specific G4-DNA topology, and thus may lack discrimination among the various G4-DNA conformations. Alternatively, the groove and loop regions of G4-DNA differ from the canonical DNA duplex, and even among the G4-DNA structures; thus, may provide a better approach for structural selectivity in binding. Recent reviews have suggested that the most thermally stabilizing and selective G4-DNA ligands often have the combined features of end-stacking and groove/loop binding [12]. Specifically, these include fused aromatic polycyclic and macrocyclic ligands, with electron withdrawing atoms/groups that can be fixed in a planar conformation for effective G-tetrad stacking, and with basic side chains targeting loops and grooves for better selective G4-DNA binding and stabilization [13]. A significant example is pyridostatin (Figure 1), a G4-selective stabilizing ligand, designed as a flexible molecule, yet able to adopt a flat conformation such that it can participate in π–π interactions with the G-tetrad. At. the same time, the molecule establishes electrostatic interactions through the amino groups that are present on the side chains. Pyridostatin alters the transcription and replication of specific human genomic loci containing high G-quadruplex clustering within the coding region, which encompasses telomeres and selected genes, such as the proto-oncogene *SRC* [14]. The molecule is an excellent G-quadruplex DNA binder with high thermal stabilization (ΔTm > 30 °C). Its interaction with an exposed planar G-quartet rather than loop sequences in solutions suggests that it might recognize a very broad spectrum of G4 structures in a living cell and interact with them [15].

RHPS4 (Figure 1) is another promising and potent G-quadruplex stabilizing ligand. The compound is a water-soluble polycyclic fluorinated acridinium cation, which carries a net positive charge in its small acridinium ring [16]. These features enhance its affinity to G-quadruplex and its ability to penetrate heavy tumor masses [17,18]. RHPS4 forms stacking interactions by binding with high affinity to the G-quadruplex at the terminal G-tetrads, with selectivity over duplex DNA [19,20]. The treatment of various cancer cell lines and tumor xenografts revealed that RHPS4 is a potent telomerase inhibitor at sub-micromolar concentrations [21]. Additionally, it causes irreversible proliferation arrest after long-term culture at non-cytotoxic concentrations, and exhibits antitumoral activity in vivo [22].

Curaxin CBL0137 (Figure 1) belongs to a class of substituted carbazoles exerting anticancer activity by complex and diverse mechanisms. Recent findings have claimed the involvement of DNA binding in the curaxin antitumor activity [23,24]. The NMR studies by our group evidenced a significant binding of curaxin with the single repeat sequence of human telomeres (TTAGGGT)_4_ and with Pu22T14T23, a model of the c-myc promoter Pu22 sequence [25]. Investigations of BMH21, BA41 and CX-5461 (Figure 1), [26] provided evidence that these compounds are also effective binders of the human telomeric and oncogene promoter G-quadruplexes, such as c-MYC and c-KYT. 

Considering all of the above reported results, we were intrigued by the challenge to explore PARP1 modulation via G-quadruplex DNA targeting. In a recent paper, Sengar and coworkers [27] analyzed, by NMR spectroscopy, the G-quadruplex structure formed by a 23-nucleotide G-rich sequence (TGGGGGCCGAGGCGGGGCTTGGG (termed as TP3)), and located 125 nucleotides (nts) upstream of the transcription start site (TSS) in the PARP1 promoter. The study revealed a three-layered intramolecular (3 + 1) hybrid G-quadruplex-folding topology, in which three strands are oriented in one direction and the fourth in the opposite direction. It should be noted that this structure exhibits unique structural features, such as an adenine bulge and a GGT base triple capping structure taking place between the central edgewise loop, propeller loop, and a flanking 5′-end. This finding offered an attractive opportunity to target the PARP1 promoter with specificity. Thus, we investigated its interaction with the above reported G-quadruplex binders (curaxin, CX-5461, BMH21, BA41, RHPS4 and pyridostatin), characterized by a strong structural diversity, with the aim of defining the key molecular features for an effective binding.

## 2. Results and Discussion

To investigate the nature of the interaction of the curaxin, CX-5461, BMH21, BA41, RHPS4 and pyridostatin with the G-quadruplex (3+1) hybrid topology in the PARP1 promoter, a combination of CD, NMR and molecular modeling studies were used.

**Figure 2:**
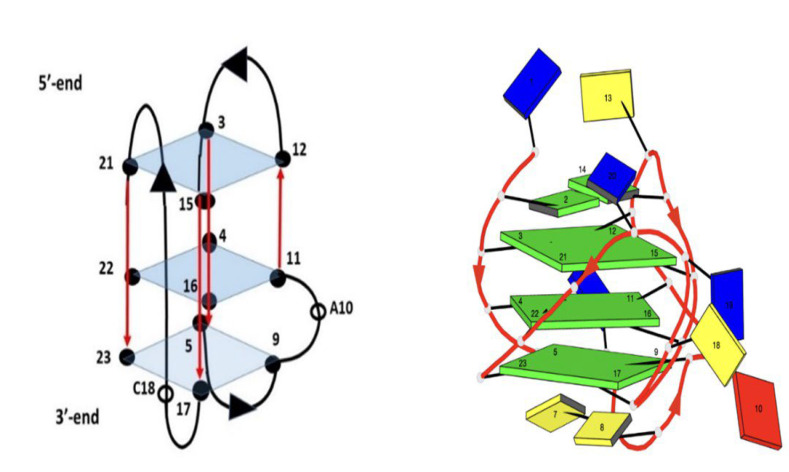
Schematic representation of TP3-T6 oligomer G-quadruplex. Adenine in red, Cytosine in yellow, Guanine in green and Thymine in blue.

To this aim, the oligonucleotide (TP3-T6), which contains a G-to-T substitution at position 6, resulting in a high-quality NMR spectrum very similar to the spectrum of TP3, was employed (Figure 2). The modified oligomer shows the formation of the same G-quadruplex-folded structure; however, having an improved temporal stability upon exposure to room temperature [27]. As reported by Sengar et al. [27], the presence of 11 well-resolved distinct peaks in the characteristic region of ^1^H NMR spectra between 10.5 and 12.5 ppm, derived from the Hoogsteen base pairs typical of G-quadruplex folding (corresponding to 12 guanines of three G-tetrad, NH of G21 and G22 being overlapped), indicate an intramolecular (3 + 1) hybrid G-quadruplex scaffold. The information concerning the mode of binding of the ligands can be obtained by analyzing the sharpening of the imino proton signals and their chemical shift variations.

The ^1^H NMR titration experiments were performed tracking the guanine imino protons of TP3-T6, by adding increasing amounts of the ligand to the oligonucleotide solution. Differences in the ligand binding mode were observed for the considered compounds.

**Figure 3:**
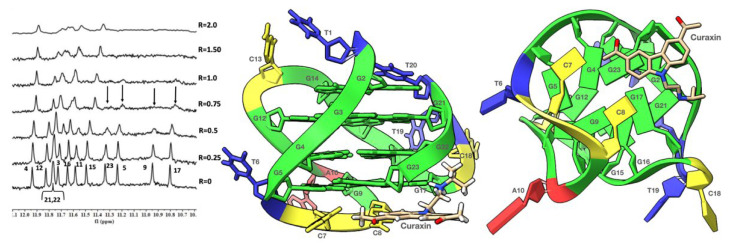
Imino proton region of the 1D NMR titration spectra of TP3-T6 with curaxin (Left), recorded at 25 °C and different R = (drug)/(DNA) ratios. The side (Center) and 3′-end (Right) views of the TP3-T6/curaxin complex obtained by Molecular Docking were created by using the Chimera-X software. In the side (Center) view, nucleotides are shown in sticks to reveal atoms and bonds, while in the 3’-end (Right) view the nucleotides are shown as slabs and filled sugars: Adenine in red, Cytosine in yellow, Guanine in green and Thymine in blue. The curaxin molecule is rendered in sticks and colored according to the atoms.

The titration of TP3-T6 with the curaxin displayed the broadening of the selected 1H imino proton signals, even at low ratio (R = (curaxin)/(TP3-T6) = 0.5). Specifically, the residues belonging to the 3′-end tetrad of the G-quadruplex G5, G9, G17 and G23 essentially disappeared at R = 1.0 (Figure 3). This behavior suggested that the curaxin mainly interacts with the 3′-end tetrad.

Adding the curaxin in excess (R > 1.50), a more significant broadening involved nearly all of the ^1^H imino proton signals. This indicates an exchange process in the intermediate regime on the NMR time scale between free and bound state, suggesting the formation of different species in solution. The disappearance of some of the imino proton signals can be explained by the loss of the hydrogen bonding, resulting in a solvent exchange. The NMR behavior demonstrated that the curaxin binds at a ratio R = 1.0, to the most accessible 3′-end tetrad of TP3-T6. Conversely, at higher ratios (R > 1.0) non-specific external interactions occur between the ligand and the oligonucleotide.

The results of the molecular docking studies confirmed the preferential interaction of the curaxin with the 3′-end tetrad (see Figure 3). The side chain is oriented towards the groove, with which it interacts mainly through a strong H-bond between the nitrogen and G17N3. On the other hand, the curaxin polycyclic system interacts with the G23 ring through a π–π stacking interaction, and with G23OP_2_ through an anion–π interaction. Despite this, the molecule fails to interact effectively with the G5-G9-G17-G23 tetrad, probably due to the steric hindrance exerted by C7 and C8.

**Figure 4:**
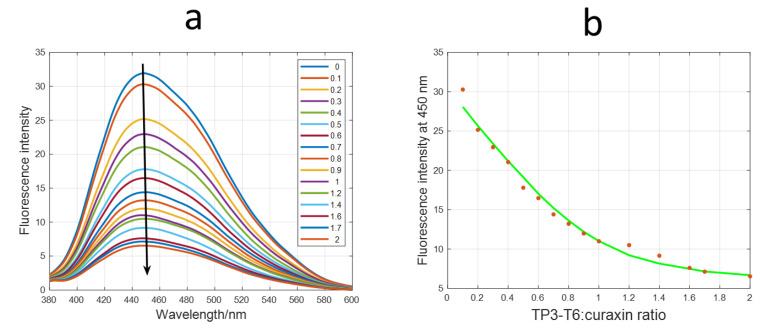
(**a**) Fluorescence spectra recorded along the titration of curaxin with TP3-T6; (**b**) Experimental (red symbols) and calculated (green line) fluorescence intensity at 450 nm for the titration of curaxin at different concentrations of TP3-T6. Conditions: 20 mM phosphate buffer (pH 7.1), 70 mM KCl of 3 μM curaxin and increasing amounts of 69 μM of TP3-T6. Excitation wavelength 334 nm.

The interaction of TP3-T6 with the curaxin was studied by molecular fluorescence spectroscopy to calculate the binding constant. The addition of TP3-T6 to a curaxin solution induced a decreased fluorescence signal intensity in the curaxin. The inverse titration of TP3-T6 with the curaxin was also performed (Supplementary Materials). In this case, an opposite effect was observed with an increased fluorescence signal with the addition of the ligand. An estimation of the stoichiometry and the binding constant (Kb) was calculated by the EQUISPEC program, starting from the multivariate analysis of the whole fluorescence spectra obtained during both of the titrations [28]. For the direct titration, the Kb value obtained by fitting with 1:1 model, was 6.72 ± 0.20 10^5^ M^−^^1^ (Figure 4). For the inverse titration, a second binding site with Kb equal to 5.63 ± 1.25 10^5^ M^−^^1^ was determined. All together, these values indicated a mild interaction of the curaxin with the TP3-T6.

**Figure 5:**
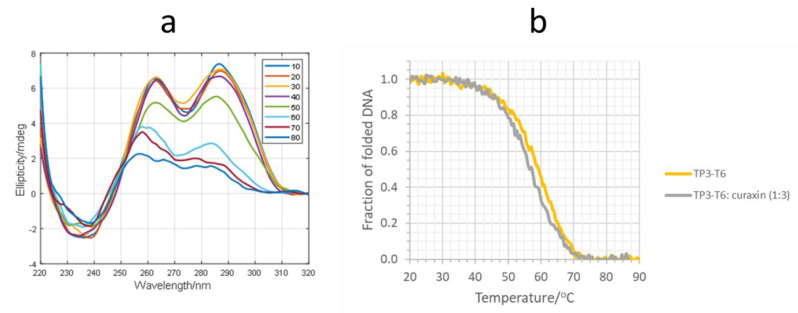
(**a**) Selected CD spectra recorded along the melting of the TP3-T6/curaxin 1:3 mixture. The whole data set is given in Supplementary Materials; (**b**) Fraction of folded DNA vs. temperature calculated from the melting of TP3-T6 and of the TP3-T6/curaxin 1:3 mixture at 265 nm. DNA and ligand concentration were 2 and 6 μM, respectively, 20 mM phosphate buffer (pH 7.1), 70 mM KCl.

CD-monitored melting experiments were used to investigate the potential stabilization of the G-quadruplex structure adopted by TP3-T6 treated with the curaxin (1:3 mixture) (Figure 5). The CD spectrum of TP3-T6 at 10 °C showed positive signals at 265 and 285 nm, which could be related to a hybrid parallel/antiparallel structure. Upon the addition of the curaxin, a small decrease in the intensity of the band at 285 nm was observed (Supplementary Materials). From the CD traces at 265 nm measured along the melting, the fraction of folded DNA was calculated, assuming a one-step process. This assumption was checked by means of multivariate analysis of the whole data set, as shown in the Supplementary Materials [29,30]. From the data in Figure 5b, it was deduced that the T_m_ values were 58.5 ± 0.8 and 56.7 ± 0.9 for TP3-T6, and for the 1:3 DNA/ligand mixture, respectively. Overall, these experiments showed small changes in the presence of the ligand, in agreement with the relatively low values of the binding constants previously determined.

All of the results did not evidence any significant stabilization of the G-quadruplex folding by the curaxin, in agreement with the external binding suggested by the NMR experiments and molecular modeling.

**Figure 6:**
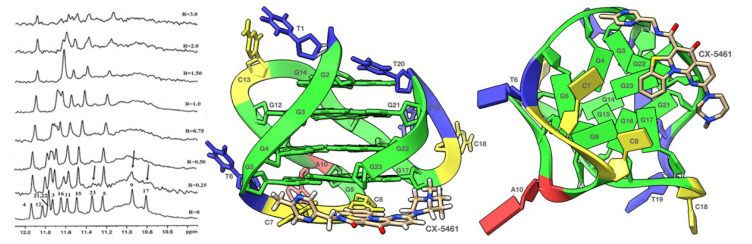
Imino proton region of 1D NMR titration spectra of TP3-T6 with CX-5461 at 25 °C at different R = (drug)/(DNA) ratios (Left). The complex between TP3-T6 and CX-5461 was obtained by molecular docking and the side (Center) and 3′-end (Right) views were created by using the ChimeraX software., nucleotides are shown in sticks to reveal atoms and bonds, the nucleotides in the side (Center) view are rendered in stick while in the 3’-end (Right) view they are shown as slabs and filled sugars. Adenine residues are colored in red, Cytosine in yellow, Guanine in green and Thymine in blue. In both views the ligand is represented as sticks and colored according to the atoms.

Similar results were found upon addition of 0.25 molar equivalent of CX-5461 to the G-quadruplex solution. A decrease in the intensity and/or a disappearance of the selected signals, such as G17, G9, G23, all belonging to the same tetrad, were observed (Figure 6). As in the previous case, at ratio R = [CX-5461]/[DNA] > 1.0, the profile of the G-quadruplex imino protons appeared altered, with a general signal broadening effect. The continuous titration to R = 2.0 stoichiometry accentuated the broadening of the imino proton resonances. The effects observed in this region suggested that the interaction of CX-5461 is with the 3′end G-tetrad (G17-G9-G23-G5). 

The complex obtained by the molecular docking between CX-5461 and the G-quadruplex confirmed the preferential interaction of the ligand with the 3′-end tetrad, as shown in Figure 6. The side chain containing the homo-piperazine ring is arranged along the groove, while the rest of the molecule lies below the G5-G9-G17-G23 tetrad. In particular, the [1,3] benzothiazolo [3,2-a]quinolin-5-one ring is quite coplanar with the 3′-end tetrad. In this orientation, the molecule establishes a network of π–π stackings with G23, as well as an anion–π interaction with the G23OP_2_ atom.

**Figure 7:**
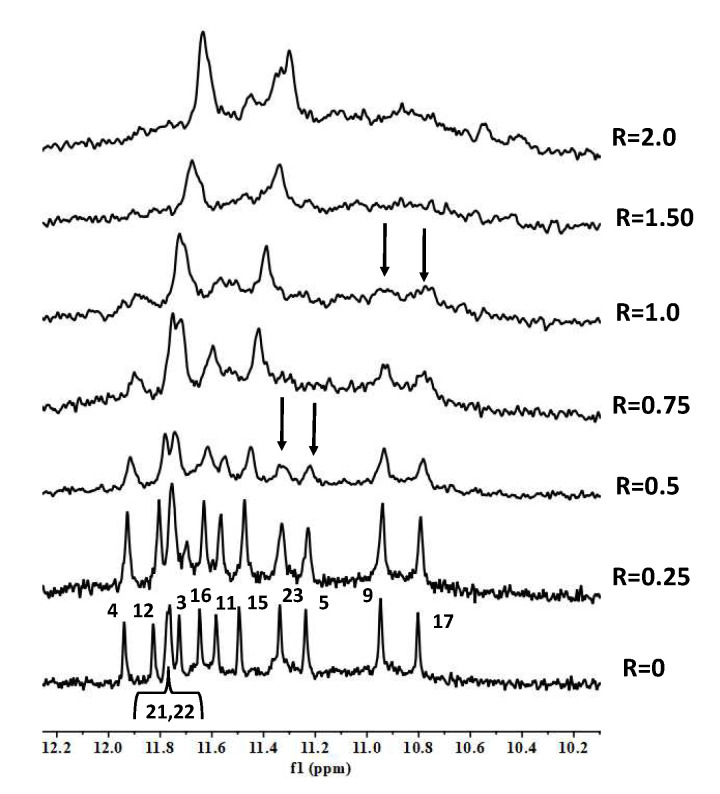
Imino proton region of 1D NMR titration spectra of TP3-T6 with BMH21 at 25 °C at different R = (drug)/(DNA) ratios.

**Figure 8:**
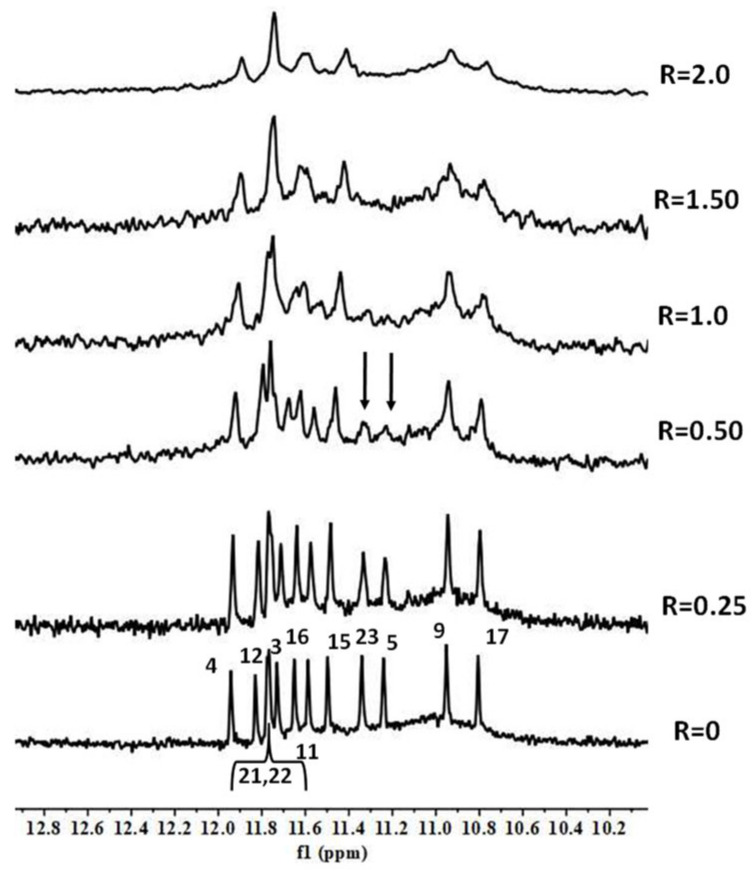
Imino proton region of 1D NMR titration spectra of TP3-T6 with BA41 at 25 °C at different R = (drug)/(DNA) ratios.

BMH21 and BA41 displayed a very similar NMR behavior. Even at a low R ratio (0.5), all of the ^1^H imino proton signals showed a significant generalized line broadening upon titration with both of the ligands, but the most affected were G5 and G23, which belong to the 3′-end tetrad (Figure 7 and Figure 8). For R > 1.0 an increased broadening was observed, likely due to an intermediate exchange regime between the free and the bound states.

These findings suggested that BMH21 and BA41 at a low ratio mainly interact with the 3′-terminal end tetrad. As in the case of the curaxin, CD-monitored titrations and melting experiments did not show any stabilization of the DNA structure upon addition of BA41 (Figure S5, Supplementary Materials).

**Figure 9:**
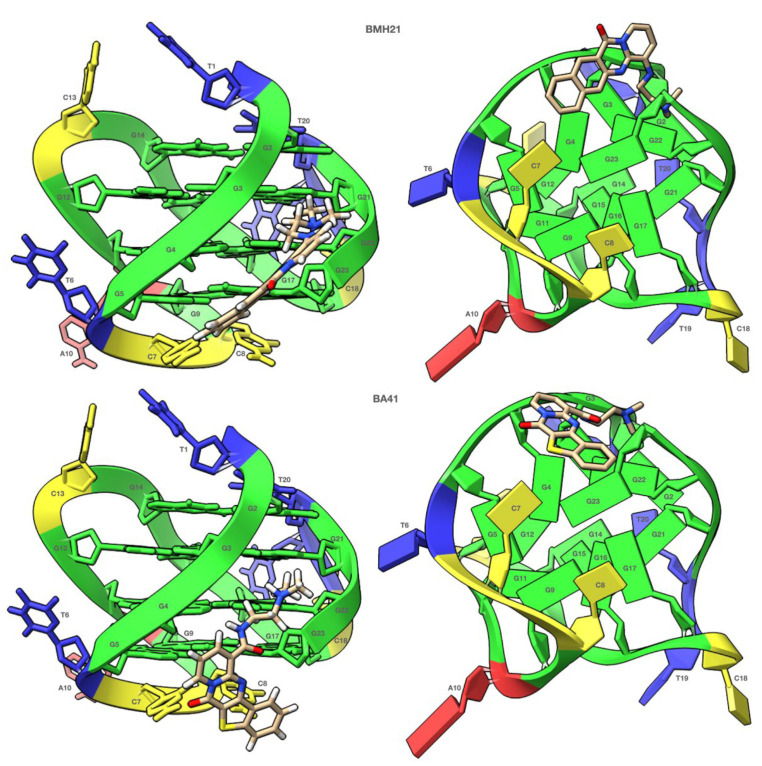
Side (Left) and 3′-end (Right) views of the complexes predicted by molecular docking of the TP3-T6 target with BMH21 and BA41. In the graphical representations, nucleotides in the side views are rendered in stick while those in the 3′-end views are rendered as slabs and filled sugars. Adenine residues are colored in red, Cytosine in yellow, Guanine in green and Thymine in blue. The ligands are always represented as sticks and colored according to the atoms. Both graphical representations were created with the ChimeraX software.

The complexes obtained by molecular docking of BMH21 and BA41 with TP3-T6 are depicted in Figure 9. The cyclic systems of BMH21 and BA41 are too bulky to allow the two molecules to fit under the 3’-end tetrad. As a result, although both of the molecules exhibit multiple interactions with the nucleotide atoms located on the groove surface, they are unable to properly stack with the planar portion of the G5-G9-G17-G23 tetrad.

**Figure 10:**
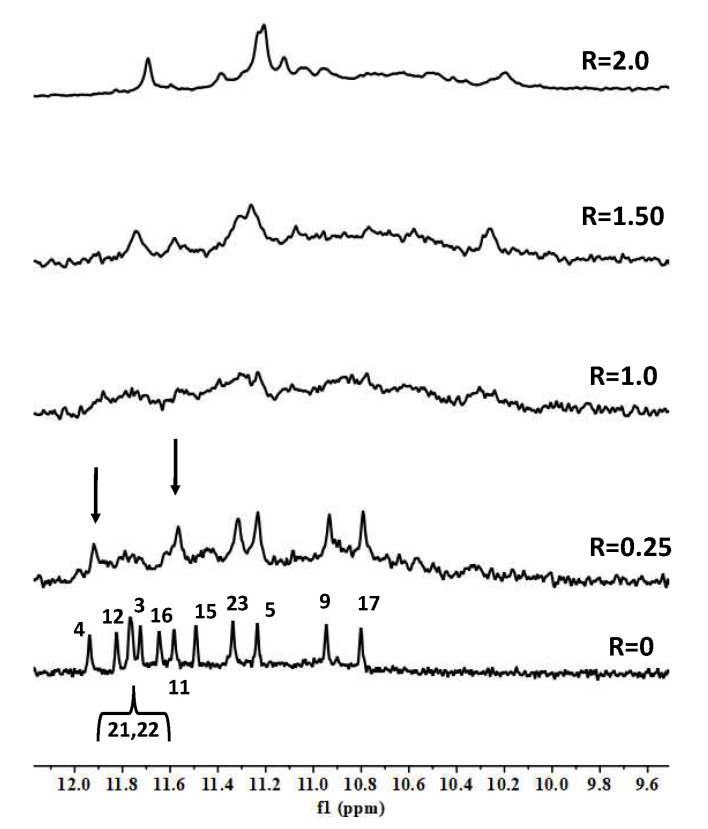
Imino proton region of 1D NMR titration spectra of TP3-T6 with RHPS4 at 25 °C at different R = (drug)/(DNA) ratios.

Concerning the titration experiments with RHPS4, we observed the broadening of selected ^1^H imino proton signals at a very low ratio (R = 0.25). In particular G3, G12, G15 and G21, all belonging to the 5′-terminal end tetrad, became very broad, until they almost completely disappeared at R = 1.0. However, not very well-defined signals were detected again at R = 2.0 (Figure 10). A molecular docking investigation did not allow to obtain an unambiguous model of interaction of RHPS4 with the oligonucleotide.

**Figure 11:**
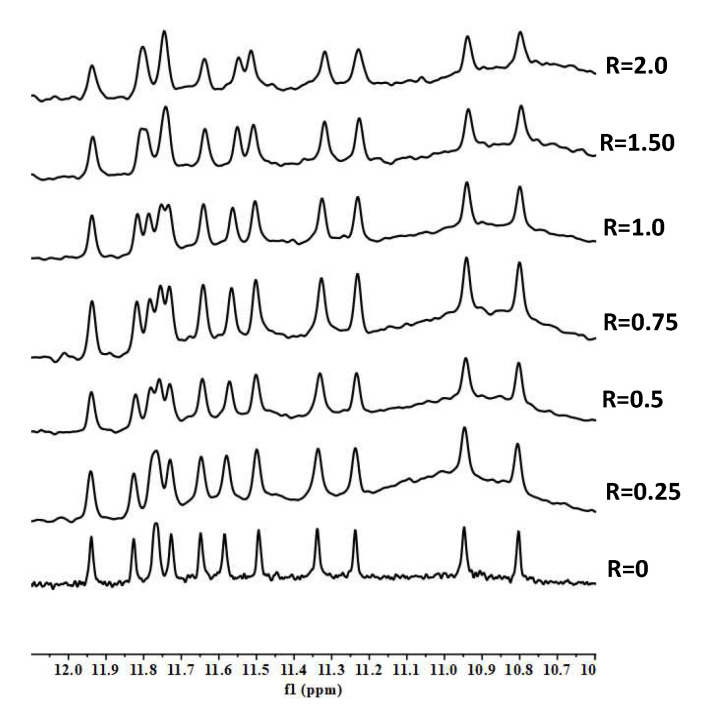
Imino proton region of 1D NMR titration spectra of TP3-T6 with pyridostatin at 25 °C at different R = (drug)/(DNA) ratios.

Upon titrating pyridostatin, minor changes were observed in the ^1^H imino protons region of NMR spectra (Figure 11). In addition, a CD-monitored melting experiment was carried out (Figure S6, Supplementary Materials). In the presence of the pyridostatin, the intensity of the CD band at 290 nm was dramatically reduced, whereas the parallel contribution remained. Upon heating, a clear stabilization of the G-quadruplex structure was observed because of the presence of the ligand, being the ΔT_m_ > 30 °C. These observations indicate a strong stabilization of the parallel contribution, as opposed to the scarce interaction with the antiparallel contribution, as already described by other authors [31]. Overall, the results highlight the importance of integrating orthogonal experimental approaches (e.g., NMR studies to point out the ligand binding mode and CD spectroscopy to describe the thermal denaturation transition, which relates the different stabilization of the folded and unfolded forms of a sequence), to define the interaction of small molecules with G4-forming sequences [32]. 

The obtained findings demonstrated that the G-quadruplex structure located at the PARP1 promoter has a peculiar structure, which makes the identification of a proper 3 + 1 stabilizing ligand a challenging goal for further investigation. The results of the molecular modeling studies gave important information, clearly showing the impossibility for most of the ligands to give effective π–π stacking interactions with the tetrad at the 5’-end. The steric hindrance deriving from the presence of G2 and G14 (as also reported by Sengar [27]) prevented ligands from effectively interacting with the G3-G12-G15-G21 tetrad. On the other side, the bases C7 and C8 only partially prevented the π–π stacking interactions with tetrad at 3 ‘-end. A summary of the interactions in the complexes between the ligands and TP3-T6 is shown in Table 1.

The NMR spectra of the curaxin complex suggested an interaction, for R = 1.0, with a specific G-tetrad residue belonging to the 3′-end. At a higher ratio, the NMR spectra provided the evidence for a groove mode of binding, which produced a degree of structural perturbation. This resulted in a destabilization of the G-quadruplex, as proved by the decease of T_m_ of the complex with curaxin. More interestingly, from our experiments it emerged that only the pyridostatin caused a stabilization of the PARP G-quadruplex structure. Pyridostatin is considered as an excellent G-quadruplex binder, mainly for its ability to adopt a flat but flexible conformation, prone to adapt to the dynamic and polymorphic nature of diverse G-quadruplex structures and for the presence of free nitrogen lone pairs [33] The evidence that only this compound was able to stabilize the PARP1 G-quadruplex confirms our previous findings about the peculiar characteristics of this structure [34], evidencing that the dimension and geometry of the ligand play a crucial role for an effective interaction. Significantly, BMH21 and BA41, which maintain a flat however small aromatic system, and an amino group in a proper position, were even able to disrupt the G-quadruplex tetrads, weakening the hydrogen bonds between the G-quartets.

## 3. Materials and Methods

*Ligands.* The compounds BMH21 and BA41 were synthesized as previously described [35]. Curaxin (CBL 0137) was purchased from Carbosynth Limited, Compton, UK. The corresponding hydrochloride was prepared by treatment with 4M HCl in dioxane.

Compound CX-5461 was purchased from ChemScene LLC, Monmouth Junction, NJ, USA. The corresponding hydrochloride was prepared by treatment with HCl in methanol. Pyridostatin and RPHS4 were kindly provided by Dr. Giovanni Beretta, Fondazione IRCCS Istituto Nazionale Tumori, Milan, Italy

*Oligonucleotides.* The synthesis of the oligonucleotides was performed by the ICTS NANBIOSIS Oligonucleotide Synthesis Platform. Unit 29. (CIBER-BBN).

*NMR.* The oligonucleotide was synthesized in 1 μmol scale on an Applied Biosystems DNA/RNA 3400 synthesizer by solid-phase 2-cyanoethylphosphoroamidite chemistry. The product obtained after the synthesis was passed through a cation exchange, Dowex 50WX2 resin, to exchange the counter ion to sodium form and then desalted with a Sephadex (NAP-10) G25 column. 

The NMR spectra were recorded on a Bruker AV600 spectrometer operating at a frequency of 600.10 MHz for ^1^H nucleus at 25 °C. The TP3-T6, 5′ –d(TGGGGTCCGAGGCGGGGCTTGGG) -3′ at 0.24–0.48 mM in G-quadruplex concentration range was prepared in 20 mM KH_2_PO_4_, 70 mM KCl, 90% H_2_O, pH = 7.0. The samples were heated to 85 °C for 1 min and then cooled at room temperature overnight. The G-quadruplex TP3-T6, 5′-d(TGGGGTCCGAGGCGGGGCTTGGG)-3′ signals were previously assigned [27]. To the TP3-T6 solution, aliquots of ligands stock solutions in DMSO-d_6_ (16–17 mM) were gradually added until R = (ligand)/(DNA) = 2.0. and ^1^H NMR spectra were recorded.

*CD and Fluorescence.* The CD spectra were recorded on a Jasco J-810 spectropolarimeter equipped with a Peltier temperature control unit (Seelbach, Germany). The DNA solution of TP3-T6 was transferred to a covered cell and ellipticity was recorded with a heating rate of approximately 0.4 °C·min^−1^. Simultaneously, the CD spectra were recorded every 5 °C from 220 to 310 nm. The spectrum of the buffer was subtracted. Each sample was allowed to equilibrate at the initial temperature for 30 min before the melting experiment began. In all of the experiments, the concentration of DNA was kept constant (2 µM) whereas the concentration of the considered ligands was increased. The medium consisted of 20 mM phosphate buffer (pH 7.1), 70 mM KCl [27].

The molecular fluorescence spectra were measured with a JASCO FP-6200 spectrofluorometer. The temperature was controlled at 20 °C, using a water bath. The fluorescence spectra were monitored using a quartz cuvette with a 10-mm path length, with the excitation and emission slits set at 10 nm, and the scan speed at 250 nm/min. The buffer consisted of 20 mM phosphate buffer (pH 7.1) and 70 mM KCl. In all of the experiments, the concentration of the ligands was 3 µM, whereas the concentration of TP3-T6 at 69 µM sequence was increased.

The determination of the ratio ligand: DNA and the calculation of the binding constants was completed from the fluorescence data recorded along the titrations of ligands with DNAs by using the EQUISPEC program [28]. This program, which is based on the multivariate analysis of the set of spectra measured along a titration, relies on the fulfillment of the law of mass action. 

The CD spectra recorded alongside the melting experiments were analyzed by means of the Multivariate Curve Resolution, based on the Alternating Least Squares procedure (MCR-ALS). This is a soft-modeling multivariate data analysis method that is not based on the fulfilment of any physico-chemical model (studies on the interactions of Ag(I) with DNA and their implication on the DNA-templated synthesis of silver nanoclusters and on the interaction with complementary DNA and RNA sequences [30]).

*Molecular modeling studies.* The first model of the NMR ensemble deposited in the Protein Data Bank (PDB accession code: 6AC7 [27]) was used to obtain the starting TP3-T6 3D-structure.

The molecular docking calculations for each of the six ligands was performed by AutoDock 4.2, [36,37] using the Lamarckian Genetic Algorithm in combination with a grid-based energy evaluation method to calculate grid maps, in a 80 Å × 80 Å × 80 Å box with a spacing of 0.01 Å. The AutoDock Toolkit (ADT) [38] was used to add the Gasteiger–Marsili charges [39] to the ligands, and the phosphorus atoms in the DNA were parameterized, using the Cornell parameters. The Addsol utility of AutoDock was used to add the solvation parameters to the system. The initial population for each molecule consisted of 100 randomly placed individuals, a maximum number of 250 energy evaluations and an elitism value of 1, a mutation rate of 0.02 and a crossover rate of 0.80. The local search was conducted applying the so-called pseudo-Solis and Wets algorithm with a maximum of 250 iterations per local search and 250 independent docking runs. The docking results were scored by using an in-house version of the simpler intermolecular energy function, based on the Weiner force field, and the lowest energy conformations (differing by less than 1.0Å in positional root-mean-square deviation (rmsd)) were collected.

The resulting complexes were placed at the center of a box (boundaries at 2.0 nm apart from all atoms) and solvated with TIP3P water molecules. Amber ff99 force field [40] with bsc1 corrections [41] was used to describe the TP3-T6 G-Quadruples. To remove the bad contacts, 1000 minimization steps were performed on the initial systems, followed by a heating ramp of short (100 ps) consecutive simulations. The production simulations consisted of 5 ns of Langevin [42,43] Molecular Dynamics (MD) NPT equilibration at 298 K and 1 atm, as implemented in NAMD [44]. During this step, all of the bonds to hydrogen atoms were constrained using the SHAKE [45] algorithm. The water molecules were kept rigid with SETTLE [46], allowing an integration time step of 0.002 ps. The electrostatic interactions were calculated using the Particle Mesh Ewald (PME) [47,48] method (Coulomb cut-off radius of 1.2 nm). A Berendsen thermostat (coupling time of 0.1ps) was applied to the systems [49]. 

The molecular graphics and analyses performed with UCSF ChimeraX, developed by the Resource for Biocomputing, Visualization, and Informatics at the University of California, San Francisco, CA, USA, with support from the National Institutes of Health R01-GM129325 and the Office of Cyber Infrastructure and Computational Biology, National Institute of Allergy and Infectious Diseases [50].

## 4. Conclusions

The enzyme PARP1 emerged as an attractive target for cancer therapy, being involved in DNA repair processes. The use of PARP1 inhibitors, which have been recently developed and approved for clinical treatments, is threatened by the rapid insurgence of resistance. Recent experiments have identified a noncanonical G-quadruplex-forming sequence within the PARP1 promoter, opening a new avenue of investigation. We were particularly intrigued by the possibility of identifying compounds acting as PARP inhibitors by an alternative approach, e.g., via G-quadruplex DNA targeting. 

One major task for the design of DNA ligands is not only the discrimination of the G-quadruplex vs. other DNA structures such as double or triple helix, but also the selectivity for a unique quadruplex topology. Considering that no PARP promoter modulator has been identified so far, we investigated the interaction of known G-quadruplex binders with the G-quadruplex-forming sequence within the PARP1 promoter.

The results obtained by NMR, CD and fluorescence titration emphasized that the structural requirements for the interaction are quite strict. In fact, compounds that strongly stabilize G-quadruplex structures had only a low affinity to the PARP1 promoter. The molecular modeling studies suggested the impossibility for most of the compounds to favorably interact with the 3′-end tetrad of this particular G-quadruplex structure. The position of the cytosine residues, C7 and C8, prevented an optimal interaction with G5 and G6, and partially also with G17. The ligands were therefore forced to interact mainly with the phosphate groups located on the surface of the groove. CX-5461 was capable of interacting promisingly with the G5-G9-G17-G23 tetrad. The shape of the [1,3]benzothiazolo [3,2-a]quinolin-5-one ring permitted CX-5461 to fit into the pocket delimited by C7 and C8, allowing it to effectively overlap with the planar portion of G23 and originating a dense network of π–π stacking interactions. Pyridostatin, endowed with a flat but flexible conformation, gave a strong stabilization of the parallel contribution, as opposed to the scarce interaction with the antiparallel contribution.

The investigated molecules can serve as molecular tools toward the identification of reliable selective PARP G-quadruplex modulators, which could serve as an alternative therapeutic approach for the regulation of PARP1 activity.

## Figures and Tables

**Table 1:** Table summarizing the main interactions observed in the TP3-T6/Ligands complexes as obtained by molecular modeling. The table shows the nucleotides involved in the interactions together with the type of interactions.

Ligands	TP3-T6 3′-End Tetrad			TP3-T6 Groove						
	G4	G17	G23	G3	G4	G17	C18	G21	G22	G23
Curaxin	-	-	PP, AP	-	-	HB	-	-	-	-
CX-5461	-	-	PP, AP	-	-	-	-	-	-	-
BMX21	AP	-	HB	SB	-	-	-	-	-	HB
BA41	-	-	HB, PP	HB, AC	AC	-	-	-	-	-
RPHS4	-	-	-	-	-	-	-	-	AC, AP	-
Pyridostatin	-	HB	PP	-	-	-	HB, AP	SB	AP	-

Interaction Types: HB (Hydrogen Bond), AC (Attractive Charge), SB (Salt Bridge), AP (Anion-π), PP (π-π).

## Data Availability

Not applicable.

## Supplementary Materials

The following supporting information can be downloaded at: https://www.mdpi.com/article/10.3390/molecules27154792/s1, Figure S1: (a) Fluorescence spectra recorded along the inverse titration of TP3-T6 with curaxin. (b) Experimental (red symbols) and fitted (green line) fluorescence intensities at 450 nm for the titration of TP3-T6 at different concentration of curaxin; Figure S2: Complete set of CD spectra recorded along the melting of TP3-T6 (a) and of the TP3-T6:curaxin (1:3) mixture (b); Figure S3: Results of the analysis by means of MCR-ALS of the complete set of CD spectra recorded along the melting of TP3-T6; Figure S4: CD spectra measured along the titration of TP3-T6 with BA41. Figure S5: Melting experiments with BMH-21 and BA41; Figure S6: CD-monitored titration with PDS.

## Sample Availability

Samples of the compounds tested are available from the authors.
