# Supplementary material for: Exploring the Interaction of G-quadruplex Binders with a (3 + 1) Hybrid G-quadruplex Forming Sequence within the PARP1 Gene Promoter Region
Source: Molecules. 2022 Jul 26;27(15):4792. doi: 10.3390/molecules27154792 (PMC9369712; doi:10.3390/molecules27154792)
Supplement: Supplementary file 1 [file molecules-27-04792-s001.zip › molecules-1810471-supplementary.pdf]

**Exploring the Interaction of G-quadruplex binders with a (3 + 1) hybrid G-quadruplex forming sequence within the PARP1 gene promoter region**

Stefania Mazzini [1,*], Salvatore Princiotto [1], Roberto Artali [3], Loana Musso [1], Anna Aviñó [4], Ramon Eritja [4], Raimundo Gargallo [5] and Sabrina Dallavalle [1,2]

[1] Department of Food, Environmental and Nutritional Sciences (DEFENS), University of Milan (Università degli Studi di Milano), 20133 Milan, Italy; stefania.mazzini@unimi.it (S.M.); salvatore.princiotto@unimi.it (S.P.); loana.musso@unimi.it (L.M.); sabrina.dallavalle@unimi.it (S.D.);
[2] National Institute of Fundamental Studies, Kandy 20000, Sri Lanka
[3] Scientia Advice di Roberto Artali, 20832 Desio, Italy; roberto.artali@scientia-advice.com (R.A.)
[4] Institute for Advanced Chemistry of Catalonia (IQAC), CSIC, Networking Center on Bioengineering, Biomaterials and Nanomedicine (CIBER-BBN), ISCIII 08034 Barcelona, Spain; aaagma@cid.csic.es (A.A.); recgma@cid.csic.es (R.E.).
[5] Department of Chemical Engineering and Analytical Chemistry, University of Barcelona, 08028 Barcelona, Spain; raimon_gargallo@ub.edu (R.G.)

* Correspondence: stefania.mazzini@unimi.it

## Table of contents:

**Figure S1. Fluorescence-monitored titration with curaxin** S2

**Figure S2. CD-monitored titration of mixtures with curaxin** S3

**Figure S3. MCR-ALS analysis** S4

**Figure S4. CD-monitored titration with BA41** S5

**Figure S5. Melting experiments with BMH-21 and BA41** S6

**Figure S6. CD-monitored titration with PDS** S7

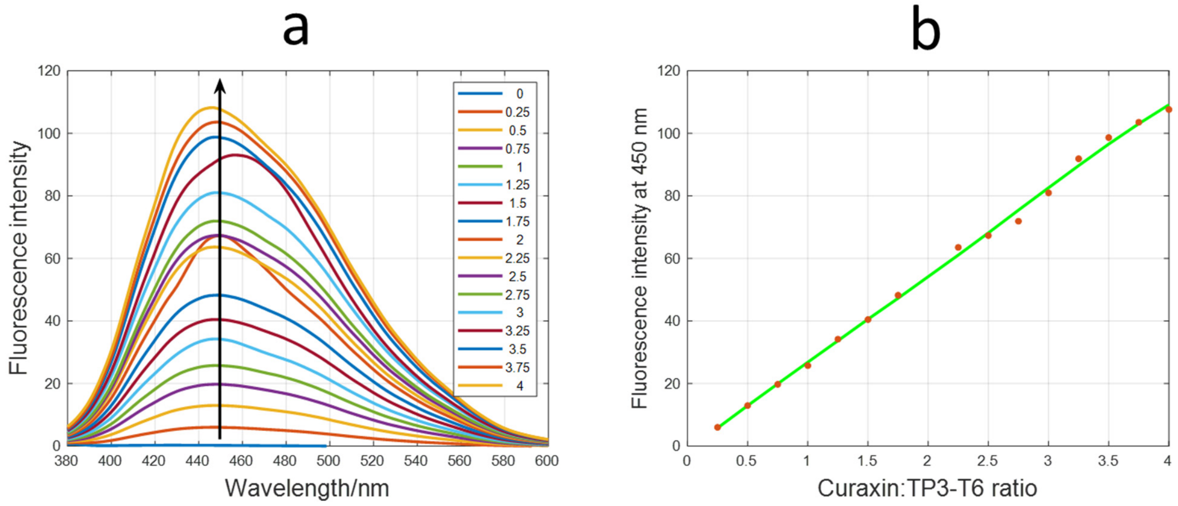


**Figure S1.** (a) Fluorescence spectra recorded along the inverse titration of TP3-T6 with curaxin; (b) Experimental (red symbols) and fitted (green line) fluorescence intensities at 450 nm for the titration of TP3-T6 at different concentration of curaxin. Conditions: 20 mM phosphate buffer (pH 7.1), 70 mM KCl of 2.41 μM TP3-T6 and increasing concentrations of 376.3 μM of curaxin.

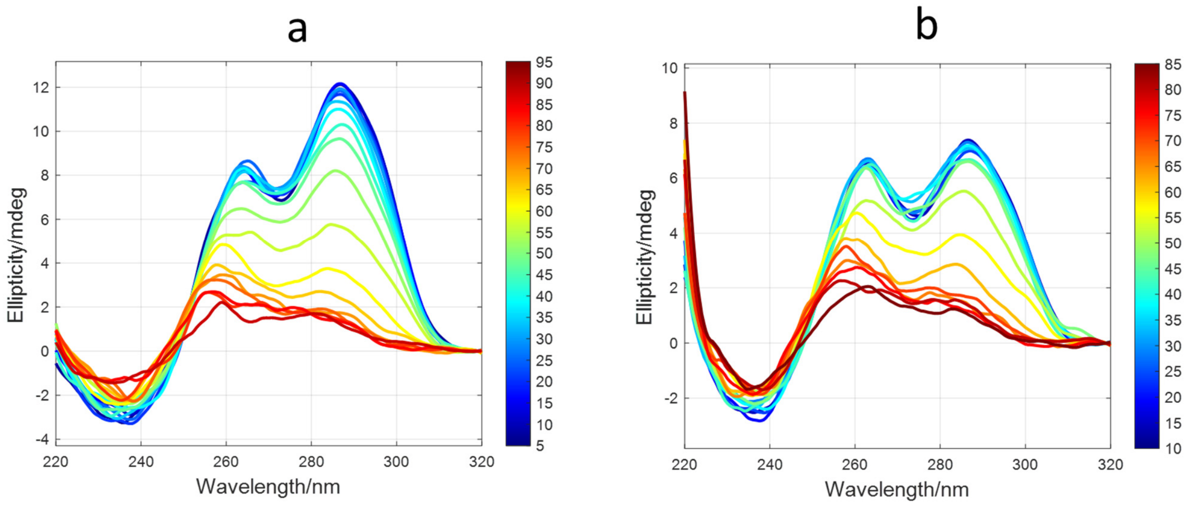


**Figure S2.** Complete set of CD spectra recorded along the melting of TP3-T6 (a) and of the TP3-T6:curaxin (1:3) mixture (b). DNA and ligand concentration were 2 and 6 µM, respectively, 20 mM phosphate buffer (pH 7.1), 70 mM KCl.

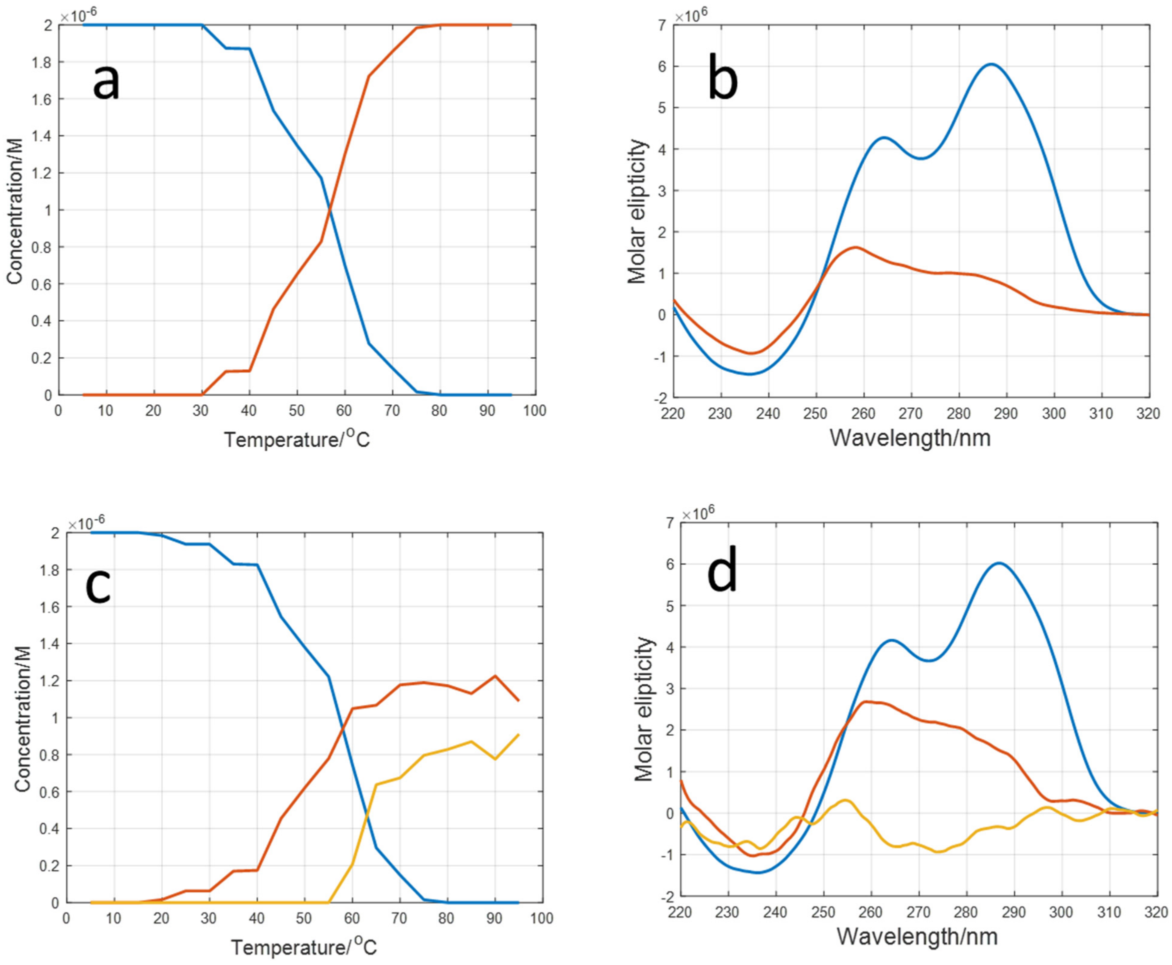


**Figure S3.** Results of the analysis by means of MCR-ALS of the complete set of CD spectra recorded along the melting of TP3-T6. (a) and (b) represent the concentration profiles and pure spectra calculated when two components were considered; (c) and (d) represent the concentration profiles and pure spectra when three components were considered. The shape of the profiles calculated in (c) for the species red and orange do not follow a logical behavior in melting experiments, as they should evolve along the melting. In addition, the decrease in the lack of fit when going from two (5.9%) to three (4.1%) components has not been considered an important factor to support the choice of three components.

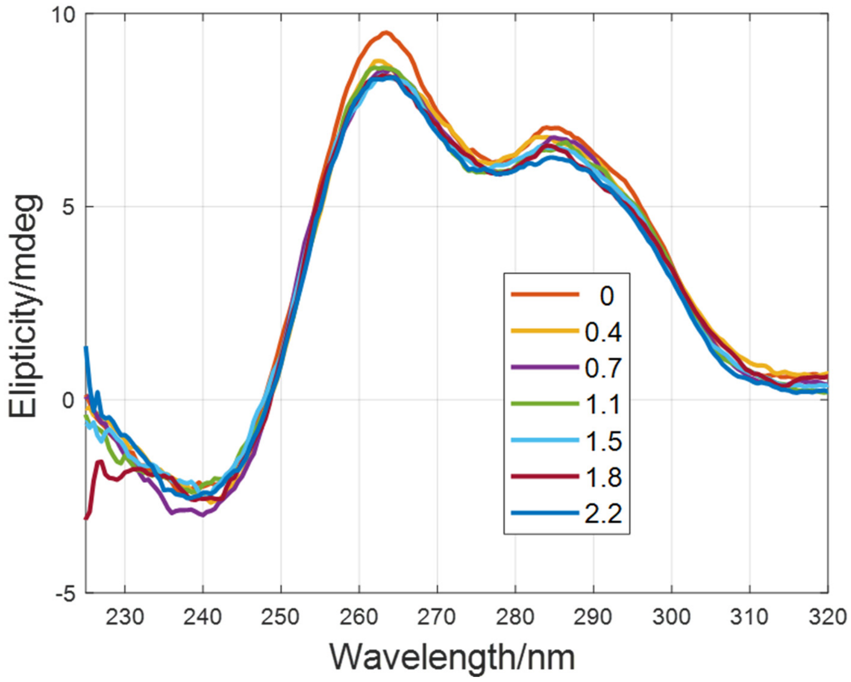


**Figure S4.** CD spectra measured along the titration of TP3-T6 with BA41. Inset show the DNA: ligand ratios. DNA concentration was 2 μM, 20 mM phosphate buffer (pH 7.1), 70 mM KCl.

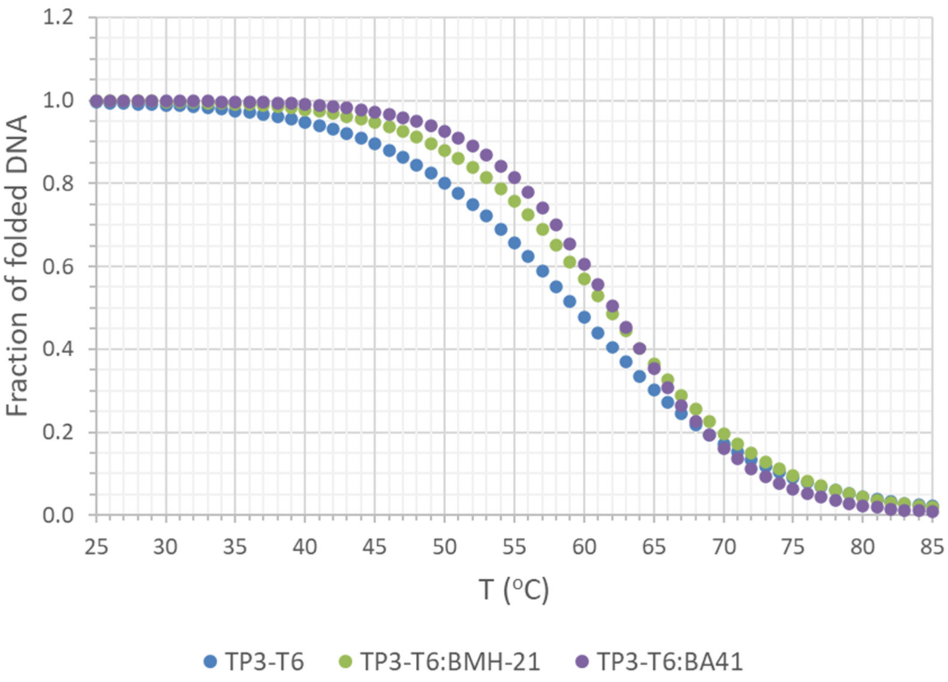


**Figure S5.** Fraction of folded DNA vs. temperature calculated from the melting of TP3-T6 and of the TP3-T6/**BMH-21** and TP3-T6/**BA41** 1:3 mixture at 265 nm. DNA and ligand concentration were 2 and 6 μM, respectively, 20 mM phosphate buffer (pH 7.1), 70 mM KCl.

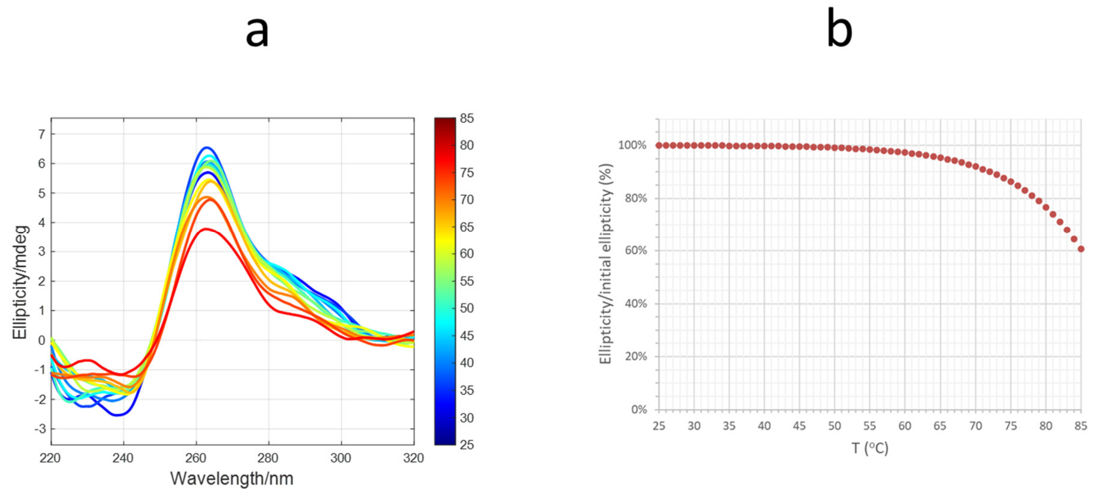


**Figure S6.** (a) CD spectra recorded along the melting of the TP3-T6/PDS 1:3 mixture; (b) Ratio of ellipticity/initial ellipticity (as percentage) vs. temperature calculated from the melting of the TP3-T6/PDS 1:3 mixture at 265 nm. DNA and ligand concentration were 2 and 6 µM, respectively, 20 mM phosphate buffer (pH 7.1), 70 mM KCl.
